# 3D printing ^18^F radioactive phantoms for PET imaging

**DOI:** 10.1186/s40658-021-00383-6

**Published:** 2021-04-28

**Authors:** Daniel Gillett, Daniel Marsden, Safia Ballout, Bala Attili, Nick Bird, Sarah Heard, Mark Gurnell, Iosif A. Mendichovszky, Luigi Aloj

**Affiliations:** 1 Department of Nuclear Medicine, Cambridge University Hospitals NHS Foundation Trust, Cambridge Biomedical Campus, Hills Road, Cambridge, CB2 0QQ UK; 2 Cambridge Endocrine Molecular Imaging Group, University of Cambridge, Addenbrooke’s Hospital, Biomedical Campus, Hills Road, Cambridge, CB2 0QQ UK; 3 Clinical Engineering, Cambridge University Hospitals NHS Foundation Trust, Cambridge Biomedical Campus, Hills Road, Cambridge, CB2 0QQ UK; 4 Clinical Pharmacology & Safety Sciences, AstraZeneca, Darwin Building, Cambridge Science Park Milton Road, Cambridge, CB4 0WG UK; 5 Metabolic Research Laboratories, Wellcome-MRC Institute of Metabolic Science, University of Cambridge, National Institute for Health Research, Cambridge Biomedical Research Centre, Addenbrooke’s Hospital, Hills Road, CB2 0QQ Cambridge, UK; 6 NIHR Cambridge Biomedical Research Centre, Addenbrooke’s Hospital, Hills Road, Cambridge, CB2 0QQ UK; 7 Department of Radiology, University of Cambridge, Cambridge Biomedical Campus, Hills Road, Cambridge, CB2 0QQ UK

**Keywords:** PET, 3D printing, Phantoms, Quality control, ^18^F

## Abstract

**Purpose:**

Phantoms are routinely used in molecular imaging to assess scanner performance. However, traditional phantoms with fillable shapes do not replicate human anatomy. 3D-printed phantoms have overcome this by creating phantoms which replicate human anatomy which can be filled with radioactive material. The problem with these is that small objects suffer to a greater extent than larger objects from the effects of inactive walls, and therefore, phantoms without these are desirable. The purpose of this study was to explore the feasibility of creating resin-based 3D-printed phantoms using ^18^F.

**Methods:**

Radioactive resin was created using an emulsion of printer resin and ^18^F-FDG. A series of test objects were printed including twenty identical cylinders, ten spheres with increasing diameters (2 to 20 mm), and a double helix. Radioactive concentration uniformity, printing accuracy and the amount of leaching were assessed.

**Results:**

Creating radioactive resin was simple and effective. The radioactive concentration was uniform among identical objects; the CoV of the signal was 0.7% using a gamma counter. The printed cylinders and spheres were found to be within 4% of the model dimensions. A double helix was successfully printed as a test for the printer and appeared as expected on the PET scanner. The amount of radioactivity leached into the water was measurable (0.72%) but not visible above background on the imaging.

**Conclusions:**

Creating an ^18^F radioactive resin emulsion is a simple and effective way to create accurate and complex phantoms without inactive walls. This technique could be used to print clinically realistic phantoms. However, they are single use and cannot be made hollow without an exit hole. Also, there is a small amount of leaching of the radioactivity to take into consideration.

## Background

Molecular imaging is a key element of many diagnostic pathways, such as oncology—using ^18^F-FDG [1], ^68^Ga-PSMA [2], ^99m^Tc-HDP [3]—and nuclear endocrinology—using ^99m^Tc-sestamibi [4], ^11^C-methionine [5–7] and ^11^C-metomidate [8, 9]. The optimal functioning of single-photon emission computed tomography (SPECT) and positron emission tomography (PET) scanners is ensured by regular quality control checks, many of which involve the use of objects called “phantoms” [10]. These phantoms need to be radioactive and are either made with long-lived radionuclides (such as ^57^Co or ^68^Ge) and supplied by commercial companies as sealed sources or have unsealed short-lived radionuclides added to water-fillable voids. Both types of phantoms usually comprise simple geometrical shapes containing one or more radioactive concentrations. The purpose of these phantoms is to check the performance of the scanners, but they are not as useful when optimising clinical imaging protocols. This optimisation is either done directly on patient images or by imaging phantoms that approximate patient anatomy. Traditionally, phantoms are made up of fillable moulded shapes containing activity distributions typically seen in clinical scans, but they do not usually replicate the complex shapes found in the human body. Recent developments in 3D printing have made it easier than ever to create more realistic phantoms [11].

3D printers have already been used to create fillable voids of replicate human anatomy [12, 13]. This technique has the advantage of being able to fashion the voids into any 3D-printable shape, and it can be used to create patient-specific phantoms. The phantom voids are filled with radioactive materials in a liquid state (such as water), and this, in turn, requires the shape to have a solid wall. However, this inactive wall affects the signal in the resulting images due to the partial volume effects and tracer displacement. Although the effect is insignificant when objects are large, it becomes very important when the modelled object of interest is small, due to the inherent spatial resolution of the imaging systems. Because of this, alternatives to fillable voids have been used to create objects without inactive walls.

These objects without inactive walls have been made using malleable materials or moulds and created using a range of materials such as wax [14] and gelatin [15]. Despite having the advantage of having no inactive walls, they are usually simple geometric shapes and, as with traditional phantoms, do not mimic human anatomy very well. However, two recent studies utilised resin-based 3D printing to create radioactive phantoms that had no inactive walls. In this work by Läppchen et al. [16], they demonstrated that resin could be labelled with ^99m^Tc and uniformly printed in 2D and 3D using a radiochromatogram scanner to scan a bar phantom and using a gamma camera to image a sphere respectively. Gear et al. [17] took this work further and demonstrated that quality control phantoms could be printed and used for routine testing of gamma cameras. In our work, we explored the feasibility of creating resin-based 3D-printed phantoms using the PET radionuclide ^18^F. In particular, we were interested in creating radioactive phantoms which would be difficult or impossible to create using a fillable void or mould.

## Methods

### Radioactive 3D-printing technique

**Fig. 1:**
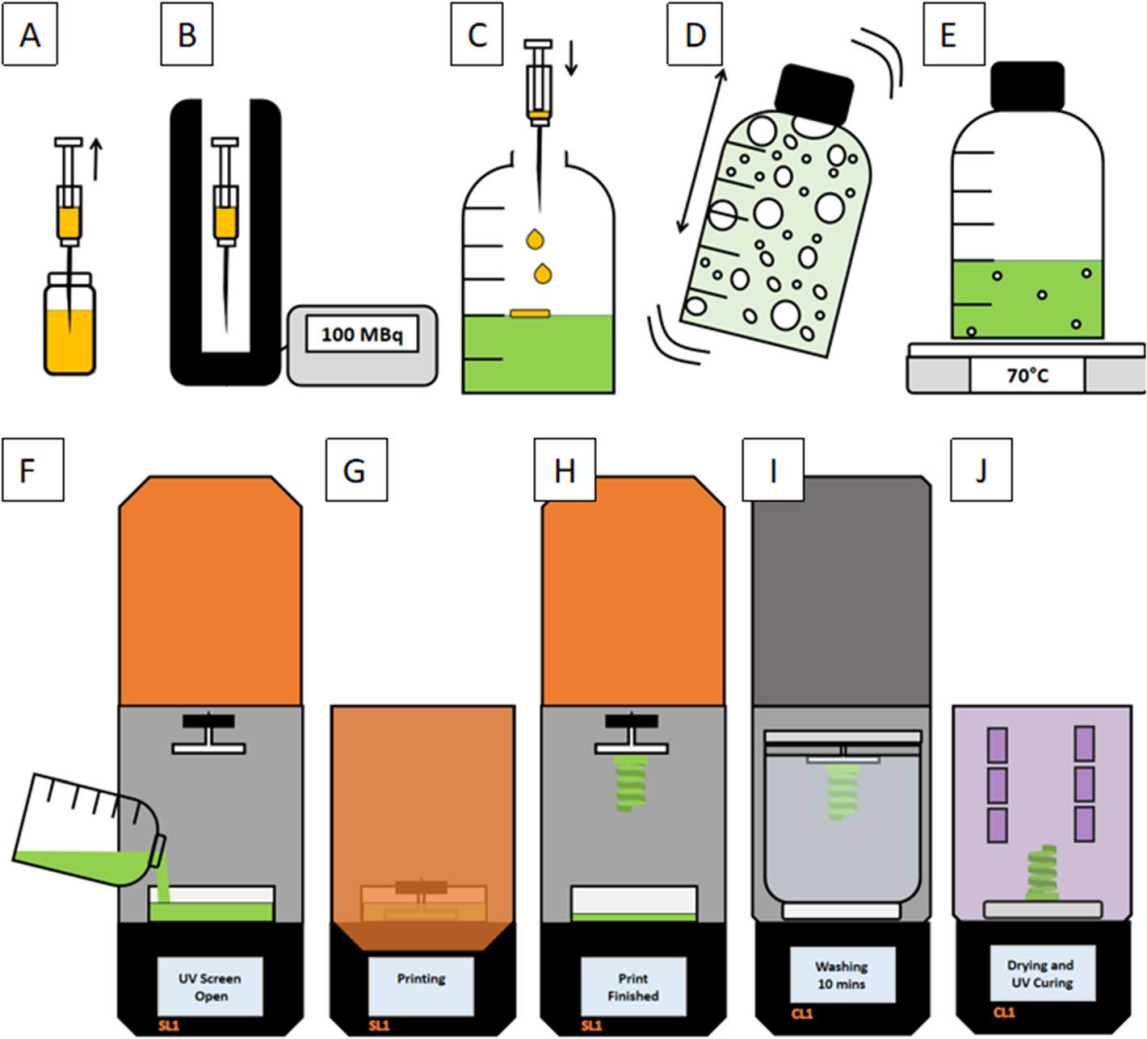
^18^F-FDG is drawn up into a syringe (**a**) and assayed using a radionuclide calibrator (**b**). The required amount of 3D-printing resin and the activity are added to a volumetric bottle (**c**). The bottle is sealed and vigorously shaken for 10 s (**d**). The bottle is placed on a heating plate for 10 min to prepare the resin for printing by helping to remove the bubbles (**e**). The radioactive resin is added to the printer (**f**), the UV protective cover is closed (**g**), and the print is started. When the print is finished (**h**), the build plate is transferred to the lid of the IPA cleaning tank (**i**), and printed objects are cleaned for 10 min. After the washing, the object is removed from the build plate and then dried using hot air and cured with UV radiation for 5 min each (**j**).

To create the radioactive resin, an emulsion of the resin and ^18^F-FDG was obtained by vigorously mixing the two together. In preparation for this, approximately 200 MBq was drawn up by hand using a shielded syringe and assayed using a radionuclide calibrator (CRC15R, Capintec, Mirion Technologies, NJ, USA). The amount of radioactivity required was estimated based on the duration of the steps involved prior to imaging to enable imaging with approximately 200 kBq/ml (typical activity concentrations seen in clinical practice are 2 to 200 kBq/ml). The activity was dispensed onto the surface of 100 ml of UV-cured resin (Prusa Research, Prague, Czech Republic) using the same shielded syringe. The container was shaken by hand vigorously for 10 s, and the heated plate was set to 70 °C to remove air bubbles by gently heating the radioactive resin. For each print created for this study, the radioactive resin emulsion was then poured into the resin tank of the masked stereolithography (SLA) 3D printer (SL1, Prusa Research, Prague, Czech Republic), and the printing was started (Fig. 1).

At the end of the printing process, the excess resin was removed by washing the object in isopropyl alcohol (IPA). Afterwards, the object was removed from the build plate, air dried and then cured with UV light for 5 min (CL1 Curing and Washing Machine, Prusa Research, Prague, Czech Republic).

### Radioactive concentration uniformity

**Fig. 2:**
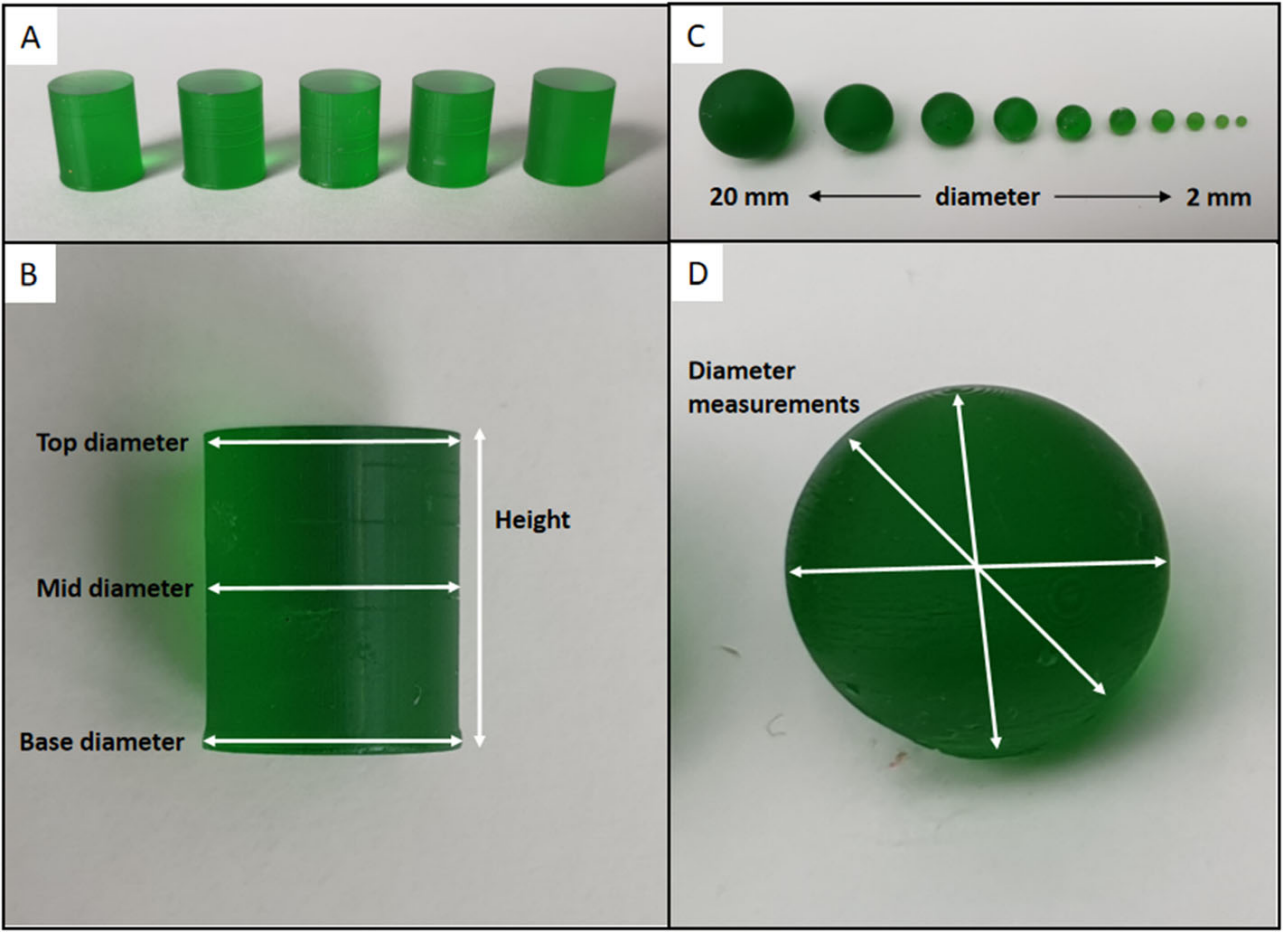
Example of 5 out of the 20 printed cylinders (**a**) and the dimensions that were measured on each cylinder (**b**). The printed spheres had nominal diameters of 20, 15, 12, 10, 8, 6, 5, 4, 3 and 2 mm (**c**) which were measured after printing at multiple orientations (**d**)

Using Fusion 360 (Autodesk, CA, USA), a cylinder with a 10-mm height and 8-mm diameter was created and exported as an STL file. The object was prepared for printing using PrusaSlicer (Prusa Research, Prague, Czech Republic) using print settings of an initial layer exposure time of 36 s, subsequent layer exposure times of 8 s, and a layer height of 0.1 mm. This cylinder was printed twenty times using the radioactive resin (Fig. 2a) to check the uniformity of the radioactive emulsion. The cylinders took 24 min to print and were imaged on a PET/CT scanner (Discovery 690 PET/CT scanner, GE Healthcare, Chicago, IL, USA). The images were reconstructed using ordered subset expectation maximisation (OSEM) iterative reconstruction using 2 iterations and 24 subsets, time-of-flight (TOF), attenuation correction (AC) and a 2-mm Gaussian filter to mimic clinical practice. To analyse the uniformity from the cylinders, twenty spherical volumes of interest (VOI), with a fixed diameter (29 mm), were centred at the maximum point within each cylinder. From these, the mean, maximum and total signal were extracted, and the coefficients of variation (CoV) calculated and used as a measure of the uniformity.

After imaging, the cylinders were measured for 300 s in a sample counter (Wizard 2480 gamma counter, Wallac). The counts were background and decay corrected, and then, a mean, standard deviation, and maximum and minimum counts were used to assess uniformity.

### Test objects

**Fig. 3:**
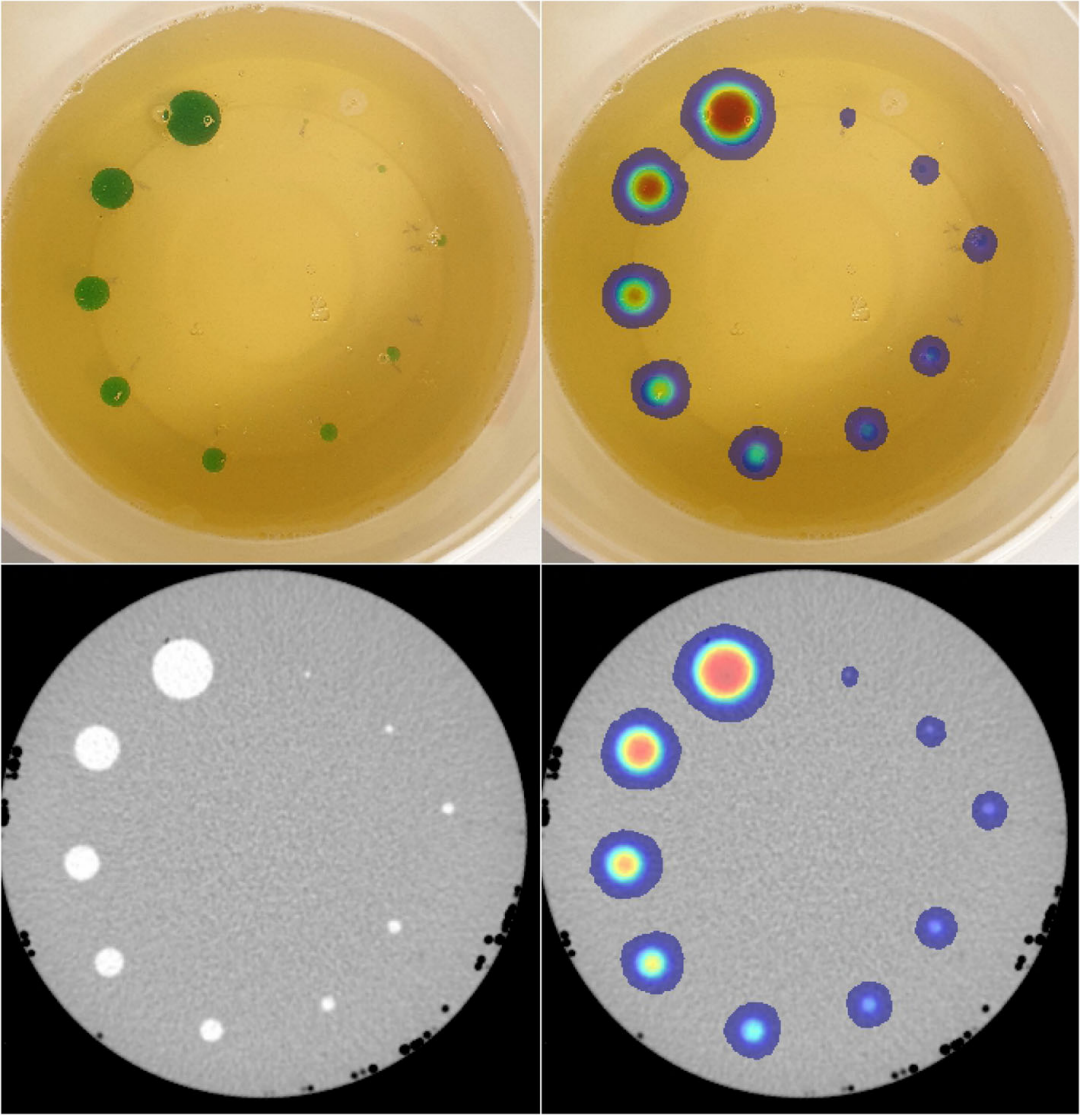
Spheres in gelatin mixture (**a**), spheres in gelatin mixture with PET signal overlaid (**b**), CT of spheres (**c**) and PET/CT images of spheres (**d**)

Using Fusion 360, a set of test spheres with diameters of 2, 3, 4, 5, 6, 8, 10, 12, 15 and 20 mm (Fig. 2c) were created and exported as STL files before preparing for printing using PrusaSlicer. These spheres were chosen to test the printing technique’s ability to produce small, well-defined objects that do not have inactive walls. The print settings were the same as for the cylinders. All of the spheres were printed at the same time and took 43 min, and after printing, they were imaged on the PET/CT scanner within a firm jelly to hold them in position and negate the need for support structures (Fig. 3a, b). To make the jelly, 60 g of powdered gelatin was added to 300 ml of cold water and heated to approximately 40°C until it all dissolved. The heated solution was poured into a cylinder and left to set for 30 min in a freezer. Afterwards, the spheres were set half into the surface of the jelly and then covered with more jelly; once again, the jelly was left for 30 min to set in the freezer. The spheres were imaged for 10 min and reconstructed using OSEM 2 iterations and 24 subsets, AC, TOF and a 3.2-mm Gaussian post filter. For comparison, the spheres of a NEMA PET IQ phantom [18] were filled with approximately 170 kBq/ml at the time of imaging and were imaged with a cold background. The imaging time and reconstruction parameters were exactly the same as used for the printed spheres. The recovery coefficients (activity concentration observed divided by the known activity concentration) of the printed spheres and the filled spheres in the NEMA were computed and plotted against the sphere diameters.

A double helix (a 3D-printing calibration object—https://www.thingiverse.com/thing:2980929) was prepared for printing using PrusaSlicer and printed using the same settings as for the cylinders. This calibration object was chosen because it is a complex shape that would be more difficult to fabricate than the majority of anatomical structures that could be printed with this technique such as tumours and endocrine glands. In addition, it would be difficult to make as a void or from a mould and is a challenging test of the capabilities of the 3D printing method using the modified resin. The double helix took 194 min to print and was then mounted inside a cylinder which was then filled with water before acquiring a 10-min static acquisition in the PET/CT scanner. The images of the spheres and the helix were reconstructed using the same parameters as for the cylinders.

### Printing accuracy

Printing accuracy was assessed by taking 10 measurements of the diameter for each printed sphere (Fig. 2d) and the height and diameter of the cylinders (Fig. 2b). These measurements were carried out using calibrated callipers to find the differences between the models and the printed spheres. From the measured diameters, the volume of the spheres was calculated and compared to the volume of the models they were printed from.

### Radioactivity leaching

The amount of radioactivity that leached out of the double helix was measured by taking a 2 ml sample 3 h after the water was added to the cylinder. To ensure the leached activity was uniformly distributed, the cylinder was shaken prior to the sample being taken. Using the volume of the cylinder and the sensitivity of the gamma counter, the amount of leaching from the object was estimated.

## Results

### Radioactive 3D printing

Creating radioactive resin was relatively simple using the emulsion technique. The emulsion remained mixed for the duration of the phantom fabrication process. In this work, the time between creating the emulsion and finishing printing was approximately 6 h because one batch of radioactive resin to print all three phantoms was used. The time required to create each phantom and the initial activity required to image approximately 200 kBq/ml are in Table 1. The amounts required to fabricate the three phantoms individually and including some other more extreme options have also been included. The minimum initial volume of resin that can be used for printing is 50 ml, and the maximum height that can be printed is 150 mm.

**Table 1:** Time required to create each phantom and the initial activity required to image approximately 200 kBq/ml

Stage	Initial activity (MBq)	Initial volume of resin (ml)	Preparation time (min)	Printing time (min)	Post processing and phantom preparation time (min)	Total time (min)
Spheres, cylinders, double helix	199	100	45	260	60	365
Spheres (max diameter 20 mm)	42	100	15	43	60	118
Cylinders (height 10 mm)	28	100	15	24	15	54
Double helix (height 95 mm)	82	100	15	193	15	223
Spheres (max diameter 2 mm)	17	50	15	8	60	83
Spheres (max diameter 37 mm)	26	50	15	78	60	153
Tumour (height 150 mm)	320	200	15	300	15	330

### Radioactive concentration uniformity

**Fig. 4:**
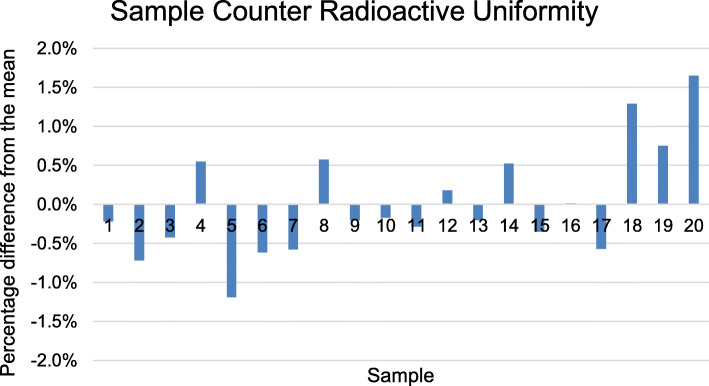
Chart showing the percentage differences from the mean counts for the cylinders acquired by the sample counter

All of the cylinders used for the uniformity assessment were printed at the same time in 24 min. They were imaged using the PET/CT scanner and measured using a sample counter. From the PET/CT images of the cylinders, the CoV of the mean, max and total signal were calculated to be 3.6%, 3.8% and 2.6% respectively. The CoV of the counts measured from the cylinders by the sample counter was found to be 0.70% and comparable to the expected CoV based on the mean number of counts of the samples of 0.12% (assuming the expected standard deviation of the counts is approximately the square root of the counts). Figure 4 shows the radioactive uniformity as shown by each sample’s deviation from the corrected mean counts. From these measurements, the maximum deviation from the mean was found to be 1.65%.

### Printing accuracy

To assess printing accuracy, the cylinders had measurements taken of the height, base diameter, mid diameter and top diameter (Fig. 2b) which were compared to the model dimensions (height 10 mm and diameter 8 mm). The mean, standard deviation and percentage difference of the measurements were 9.92 mm (sd 0.02, Δ −0.8%), 8.27 mm (sd 0.05 mm, Δ 3.4%), 8.01 (sd 0.01 mm, Δ 0.1%) and 8.03 mm (sd 0.01, Δ 0.4%) mm for the height, base, and mid, and top diameters respectively. The data is summarised in Table 2.

**Table 2:** Differences of measured dimensions from the printing model

Printing accuracy	Height	Base diameter	Mid diameter	Top diameter
Model (mm)	*10.00*	*8.00*	*8.00*	*8.00*
Mean (mm)	9.92	8.27	8.01	8.03
SD (mm)	0.02	0.05	0.01	0.01
Difference (%)	−0.8%	3.4%	0.1%	0.4%

After printing, the spheres were imaged using the PET/CT scanner and used to assess printing accuracy. Ten measurements of the diameters of each sphere were taken using calibrated callipers and compared to the computer models. The mean differences and percentage differences were −0.074 mm (−3.7%), −0.113 mm (−3.8%), −0.129 mm (−3.2%), −0.063 mm (−1.3%), −0.037 mm (−0.6%), −0.097 mm (−1.2%), −0.033 mm (−0.3%), −0.083 mm (−0.7%), −0.095 mm (−0.6%) and −0.178 mm (−0.9%) for the 2, 3, 4, 5, 6, 8, 10, 12, 15 and 20-mm diameter spheres respectively. The data is summarised in Table 3.

**Table 3:** Sphere diameter measurements

Sphere diameter (mm)	2	3	4	5	6	8	10	12	15	20
Diameter measurement mean (mm)	1.93	2.89	3.87	4.94	5.96	7.90	9.97	11.92	14.91	19.82
Absolute difference (mm)	−0.074	−0.113	−0.129	−0.063	−0.037	−0.097	−0.033	−0.083	−0.095	−0.178
Percentage difference (%)	−3.7	−3.8	−3.2	−1.3	−0.6	−1.2	−0.3	−0.7	−0.6	−0.9

### Test objects

**Fig. 5:**
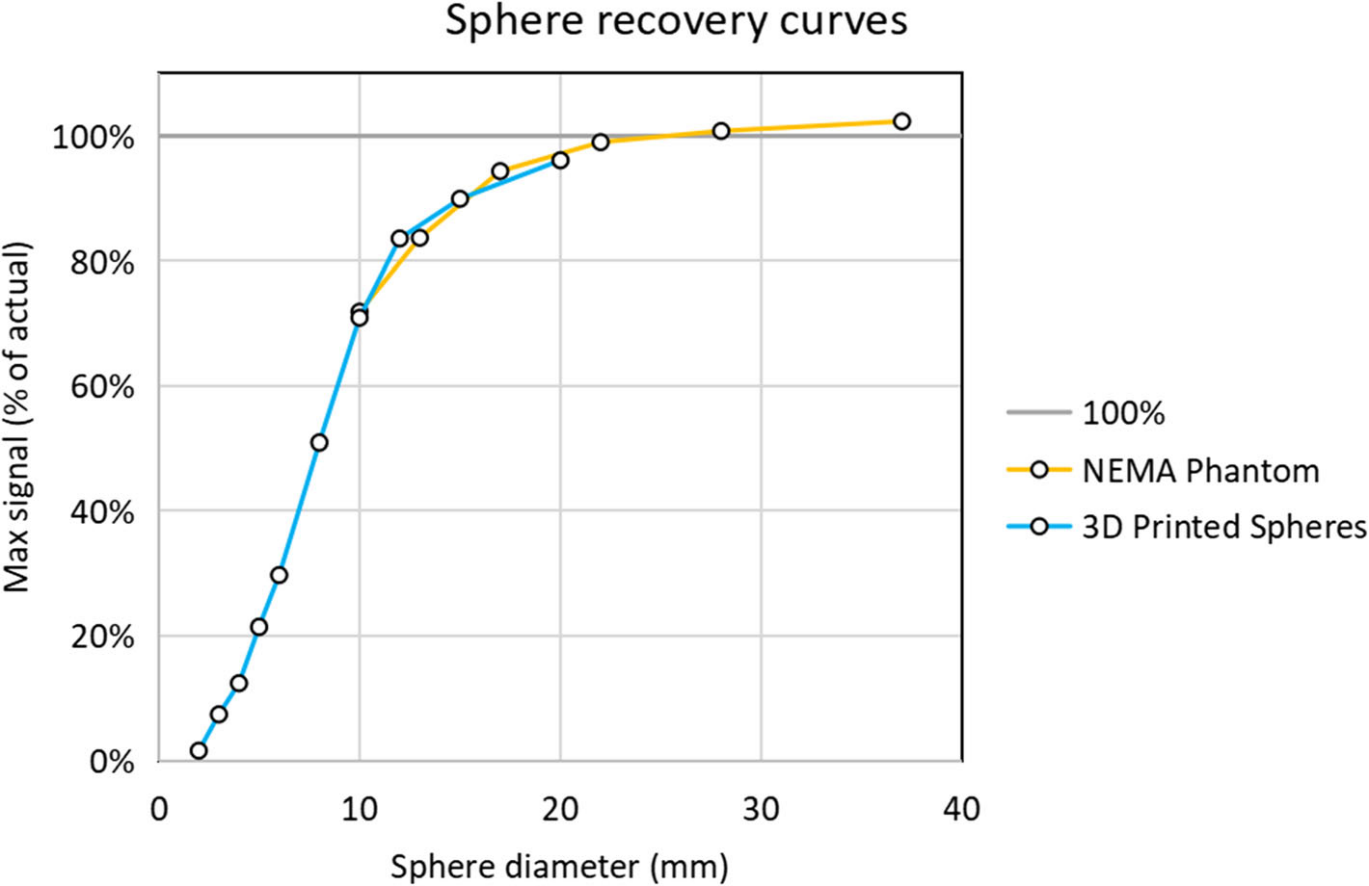
Plot of max signal from each printed sphere and each NEMA phantom sphere compared with the actual concentration. Where the sizes of the spheres are comparable between the two phantoms, the curves overlap

All of the spheres were visible on CT (Fig. 3c) and PET (Fig. 3b and d). In the reconstructed dataset, each sphere was outlined using the thresholding tool to create a VOI. The max signal within each VOI was used as a measure of recovery. Figure 5 shows a bar chart of the max signal vs the sphere diameter. As expected, due to the reconstruction algorithm, scanner limitations and the partial volume effect, there is a convergence towards the actual concentration as the spheres get larger and importantly, where the curves from the printed spheres and the fillable spheres overlap the recovery values are comparable (Fig. 5).

The helix was successfully imaged using the PET/CT scanner and appeared as expected. The base and the coils were easily visible, and the horizontal bars were not seen distinctly. Most importantly, the appearance of the radioactive concentration was uniform throughout the height of the double helix.

### Radioactivity leaching

**Fig. 6:**
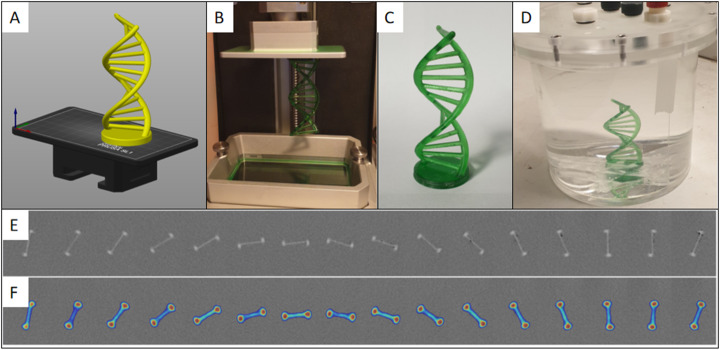
Helix model prepared for printing in PrusaSlicer (**a**). Helix after printing (**b**). Helix after removal from build plate, washed, dried and cured (**c**). Helix mounted in a cylinder of water for imaging (**d**). Axial CT slices (**e**). Axial PET/CT slices (**f**)

The amount of radioactivity that leached into the water of the phantom was 0.72% of the activity in the helix. This was calculated by taking a sample from the water at 3 h after the phantom (Fig. 6d) being in the water. This activity was not visible above the background count rate on the scanner.

## Discussion

We have, for the first time, demonstrated that radioactive PET phantoms can be created using a consumer SLA 3D printer and that it can be used to create phantoms that are complex in their shape and structure. This may allow to better mimic human anatomy and simulate heterogeneous radioactivity concentrations that are normally present in the in vivo setting.

The printing process seemed unaffected by the presence of the radioactivity and the carrier liquid; however, we still found limitations to the technique, most notably the ability to print large solid blocks (Table 4). We tried to do this to test the uniformity using the scanner and so printed a cuboid that was slightly smaller than the build plate. However, this did not produce the expected shape because the resin tends to shrink by a few microns after being cured, and when the volume is large, this causes the edges of some layers not to adhere to each other, resulting in cracks. This is a known limitation of SLA 3D printing which may have been exacerbated by the addition of radioactive carrier liquid. This means that this particular method is unsuitable for creating large solid objects. It does appear suitable, however, for generating small- to medium-sized intricate shapes that would be difficult or even impossible to make with conventional techniques such as fillable voids or moulds. Objects as small as 0.5 mm in width or diameter can be printed successfully, and although they are fragile, they may have a role in preclinical PET imaging where the spatial resolution is superior to that in clinical practice.

**Table 4:** Advantages and limitations of radioactive 3D printing technique

Advantages	Limitations
No inactive walls	Cannot print large solid objects
Accurate geometry	Phantoms are single use
Short print times	Cannot print hollow objects without an exit hole
Can print complex 3D shapes	Leaching of radioactivity into water (small amount <1%)

We were able to create phantoms using ^18^F despite its short half-life because the 3D printer used masked SLA technology. This technology uses an LCD to mask a UV light to the shape required for each layer. This means that compared to conventional SLA printing—which uses a laser point to trace each layer—it is quicker. The limiting factor is therefore the height of the object being printed, but using this technology, we were able to print the helix object (Fig. 6) which was 95-mm high in 194 min (i.e. 0.48mm/min). At this rate, an object the maximum size of the printer could be printed in 5 h, but more importantly, smaller objects such as the uniformity cylinders (Fig. 2a) and spheres, up to 20 mm in diameter, (Fig. 2c) can be printed in just 24 min and 43 min respectively, no matter how many there are.

An important consideration when carrying out this work is the radiation dose to the operator. Overall, the procedure is estimated to result in whole body and extremity doses of 0.01 mSv and 2.7 mSv respectively. The activities that cause the highest doses are shaking the bottle to create the emulsion and removing the printed object from the build plate. The dose from shaking the bottle could be reduced if the resin and radioactivity were mixed using a mechanical device such as magnetic stirrer. The dose from removing the printed object from the build plate could be reduced if object could be removed with tools or removed more easily by using a flexible build plate instead of the standard rigid one. Both potential dose-reduction strategies will be explored.

We were able to show, just like Gear et al. [17], that objects printed using these techniques are uniform and can be used for quality control (Fig. 3). Importantly, the uniformity of the radioactivity within the test objects (Fig. 2a) was very good and more than adequate for the purposes of making phantoms that replicate typical radioactivity concentrations in patients (2 to 200 kBq/ml). This result has given confidence in the technique to use it for image optimisation instead of or in combination with water-filled phantoms.

The printed spheres were imaged in a cold background. This was done so that the smallest spheres would be visible in the reconstructed images. The results were compared with the NEMA PET IQ phantom which had a similar activity concentration and number of counts. Using a standard clinical protocol, the signal from the spheres was compared to the known activity concentrations to generate recovery curves (Fig. 5). Where the spheres were comparable in diameter, the recovery curves are similar. These curves highlight a potentially important use for this type of phantom in the development of reconstruction algorithms and partial volume correction algorithms. These algorithms can only be optimised and properly tested using phantoms with small well-defined objects. The NEMA micro-PET IQ phantom [19] and the Derenzo phantom [20] do both have smaller objects than the NEMA PET IQ phantom, having rods as small as 1 mm and 0.8 mm in diameter respectively. However, despite the availability of these phantoms, there is a role for radioactive 3D printing in creating test objects since they are comparably small but, importantly for partial volume correction and small-animal imaging, can also be of any shape and do not need an inactive wall.

We were able to print accurate spheres as small as 2 mm in diameter with a well-defined activity concentration and without an inactive wall (Figs. 2c and 4). This ability will enable phantoms to be made which mimic the anatomy and pathology seen clinically in investigations such as pituitary [5–7] and adrenal [8, 9] adenoma localisation, that has not been possible before. They could also play a role in the development of partial volume correction (PVC) algorithms. A systematic review of the literature preceding 2017 concluded that there is still more work needed to be able to adopt PVC clinically [21]. One reason for this has been the use of traditional phantoms such as the NEMA PET phantom in designing the PVC algorithms which have been shown to overestimate PVC in cardiac PET/CT [22]. These works highlight how critical wall-free phantoms could be in optimising imaging protocols because traditional phantoms that use fillable voids have relatively thick inactive walls that cannot get close to approximating the shapes and proximity of the anatomical structures being imaged in these investigations. Another area where wall-free phantoms could play an important role is in the development of new image reconstruction techniques. Novel techniques are being developed all the time [23, 24], and it is essential that the imaged object has a well-defined shape and known activity concentration. Often, this requirement is met by using traditional phantoms, but the potential to use more complex geometries such as those seen in clinical practice (tumours [2, 25] and adrenal glands [9]) will potentially enable better algorithms to be developed by being more life-like or even more challenging.

The printing accuracy was remarkably good with the maximum deviation of the model being 0.27 mm (3.4%) at the bottom of the cylinder (Fig. 2). This is an effect caused by the longer exposure time for the first layer. The longer exposure is required to ensure the print is fixed securely to the build plate but also results in more resin being cured by scattered UV light. The effect is not seen as the rest of the cylinder is printed, with the mid and top diameters being within 0.01 ± 0.01 mm and 0.03 ± 0.01mm respectively. Although small, this deviation could be compensated for by adjusting the model. The small amount of shrinkage observed in the heights of the cylinders could also be adjusted for by enlarging the height of the model; however, given that the deviation in the height and the bottom diameter are both far smaller than the resolution of the scanner, it is not felt that this will have a noticeable or measurable effect on the final image.

The helix demonstrated that complex objects can be printed and imaged using a PET scanner (Fig. 6). There are no structures within the human body that could be approximated with a helix, but, nevertheless, it acted as a potential worst case scenario for the printer because it had multiple overhanging bridges and was relatively tall (95 mm). As many biological structures are smaller than this, it showed that there is real clinical potential to be gained by being able to optimise a PET scan using the radionuclide most commonly used, in the shape and concentration found in clinical practice. Examples include the pituitary gland which is small (< 10 mm) and can have microadenomas as small as 2 mm in diameter within it, and the adrenal glands can have equally small microadenomas but are larger (50 mm) and more complex in their shape (V or Y shaped).

The amount of leaching of the radioactivity into the background was relatively low (<1%) and was not visible on the PET scan, although it was detectable in an aliquot of the background (taken at 3 h) when assayed using a sample counter. The size of the phantom was large relative to the printed object and so did not represent a problem; however, more work is needed to determine whether the amount of leaching would be a problem for smaller background volumes. The surface area of the printed object is also likely to play a key part in the amount of the leaching with more leaching expected as the surface area increases. Although there is no requirement for these phantoms to be in a water-filled background, this is a potential limitation (Table 4) if this is how they are to be utilised. Future work with this technique will explore a range of background activity concentrations to evaluate the extent of the effect of the inactive walls and the partial volume effect.

It is theoretically possible to create phantoms of any printable shape and size, and as already mentioned, this included a vast range of options, but there are limitations with what is printable using this technique (Table 4). In addition to the limitations already mentioned, it is not possible to print shapes that are hollow and completely sealed. Without a hole in the hollow structure, excess resin will be captured and have no way of being removed; therefore, an exit hole must always be included in this type of structure.

## Conclusion

We have demonstrated that creating a radioactive resin emulsion is a simple and effective way to create phantoms without an inactive wall that can be imaged using a PET scanner. Our method is quick enough to use widely available ^18^F-FDG and could be used to create any SLA 3D printable object.

## Data Availability

The datasets used and/or analysed during the current study are available from the corresponding author on reasonable request.

## Abbreviations

AC: Attenuation correction
CoV: Coefficient of variation
CT: X-ray computed tomography
FDG: Fluorodeoxyglucose
IPA: Isopropyl alcohol
OSEM: Ordered subset expectation maximisation
PET: Positron emission tomography
SLA: Stereolithography
SPECT: Single-photon emission tomography
STL: Standard Tessellation Language
TOF: Time-of-flight
UV: Ultraviolet radiation
VOI: Volume of interest

## References

[1] Chen H, Pang Y, Wu J, Zhao L, Hao B, Wu J (2020). Comparison of [68Ga]Ga-DOTA-FAPI-04 and [18F] FDG PET/CT for the diagnosis of primary and metastatic lesions in patients with various types of cancer. Eur J Nucl Med Mol Imaging.

[2] Wester H-J, Schottelius M (2019). PSMA-targeted radiopharmaceuticals for imaging and therapy. Semin Nucl Med.

[3] Nakajima K, Edenbrandt L, Mizokami A (2017). Bone scan index: a new biomarker of bone metastasis in patients with prostate cancer. Int J Urol Off J Jpn Urol Assoc.

[4] Redman S, Graham R, Little D (2019). Parathyroid scintigraphy. Nucl Med Commun.

[5] Koulouri O, Kandasamy N, Hoole AC, Gillett D, Heard S, Powlson AS (2016). Successful treatment of residual pituitary adenoma in persistent acromegaly following localisation by 11C-methionine PET co-registered with MRI. Eur J Endocrinol.

[6] Koulouri O, Hoole AC, English P, Allinson K, Antoun N, Cheow H (2016). Localisation of an occult thyrotropinoma with 11 C-methionine PET-CT before and after somatostatin analogue therapy. Lancet Diabetes Endocrinol.

[7] Bashari WA, Senanayake R, Fernández-Pombo A, Gillett D, Koulouri O, Powlson AS (2019). Modern imaging of pituitary adenomas. Best Pract Res Clin Endocrinol Metab.

[8] Hennings J, Sundin A, Hägg A, Hellman P (2010). 11 C-metomidate positron emission tomography after dexamethasone suppression for detection of small adrenocortical adenomas in primary aldosteronism. Langenbeck's Arch Surg.

[9] O’Shea PM, O’Donoghue D, Bashari W, Senanayake R, Joyce MB, Powlson AS (2019). 11C-Metomidate PET/CT is a useful adjunct for lateralization of primary aldosteronism in routine clinical practice. Clin Endocrinol.

[10] Madsen MT, Sunderland JJ, DeWerd LA, Kissick M (2014). Nuclear medicine and PET phantoms. The phantoms of medical and health physics: devices for research and development.

[11] Filippou V, Tsoumpas C (2018). Recent advances on the development of phantoms using 3D printing for imaging with CT, MRI, PET, SPECT, and ultrasound. Med Phys.

[12] Gear JI, Long C, Rushforth D, Chittenden SJ, Cummings C, Flux GD (2014). Development of patient-specific molecular imaging phantoms using a 3D printer. Med Phys.

[13] Robinson AP, Tipping J, Cullen DM, Hamilton D, Brown R, Flynn A (2016). Organ-specific SPECT activity calibration using 3D printed phantoms for molecular radiotherapy dosimetry. EJNMMI Phys.

[14] Bazañez-Borgert M, Bundschuh RA, Herz M, Martínez M-J, Schwaiger M, Ziegler SI (2008). Radioactive spheres without inactive wall for lesion simulation in PET. Z Med Phys.

[15] Kao YH, Luddington OS, Culleton SR, Francis RJ, Boucek JA (2014). A gelatin liver phantom of suspended 90Y resin microspheres to simulate the physiologic microsphere biodistribution of a postradioembolization liver. J Nucl Med Technol.

[16] Läppchen T, Meier LP, Fürstner M, Prenosil GA, Krause T, Rominger A, et al. 3D printing of radioactive phantoms for nuclear medicine imaging. EJNMMI Phys. 2020;7. 10.1186/s40658-020-00292-0 PMC7176799 32323035

[17] Gear JI, Cummings C, Sullivan J, Cooper-Rayner N, Downs P, Murray I (2020). Radioactive 3D printing for the production of molecular imaging phantoms. Phys Med Biol.

[18] National Electrical Manufacturers Association (NEMA) (2007). NEMA standards publication NU 2–2007: performance measurements of positron emission tomographs.

[19] National Electrical Manufacturers Association (NEMA) (2008). NEMA NU4-2008: performance measurements of small animal positron emission tomographs.

[20] Cox BL, Graves SA, Farhoud M, Barnhart TE, Jeffery JJ, Eliceiri KW (2016). Development of a novel linearly-filled Derenzo microPET phantom. Am J Nucl Med Mol Imaging.

[21] Cysouw MCF, Kramer GM, Schoonmade LJ, Boellaard R, de Vet HCW, Hoekstra OS. Impact of partial-volume correction in oncological PET studies: a systematic review and meta-analysis. Eur J Nucl Med Mol Imaging. 2017;44(12):2105–16. 10.1007/s00259-017-3775-4 PMC5656693 28776088

[22] Lance Gould K, Bui L, Kitkungvan D, Pan T, Roby AE, Nguyen TT (2020). Pitfalls in quantitative myocardial PET perfusion I: myocardial partial volume correction. J Nucl Cardiol Off Publ Am Soc Nucl Cardiol.

[23] Marquis H, Deidda D, Gillman A, Willowson KP, Gholami Y, Hioki T (2021). Theranostic SPECT reconstruction for improved resolution: application to radionuclide therapy dosimetry. EJNMMI Phys.

[24] Wadhwa P, Thielemans K, Efthimiou N, Wangerin K, Keat N, Emond E (2021). PET image reconstruction using physical and mathematical modelling for time of flight PET-MR scanners in the STIR library. Methods..

[25] Larson CR, Wiggins RH (2019). FDG-PET imaging of salivary gland tumors. Semin Ultrasound CT MRI.

